# The Oncometabolite 2-Hydroxyglutarate Is Upregulated in Post-Prostatectomy PSA Recurrence of Prostate Cancer: A Metabolomic Analysis

**DOI:** 10.3390/molecules30163316

**Published:** 2025-08-08

**Authors:** Dontrel W. Spencer Hairston, Shamira Sridharan-Weaver, Abheek Gandhi, Neelu Batra, Blythe P. Durbin-Johnson, Marc A. Dall’Era, Paramita M. Ghosh

**Affiliations:** 1 Veterans Affairs-Northern California Health System, Mather, CA 95655, USA; 2 Department of Urologic Surgery, School of Medicine, University of California Davis, Sacramento, CA 95718, USA; 3 Department of Computer Science, University of California Irvine, Irvine, CA 92697, USA; 4 Department of Biochemistry and Molecular Medicine, School of Medicine, University of California Davis, Sacramento, CA 95718, USA; 5 Department of Public Health Sciences, University of California Davis, Davis, CA 95616, USA

**Keywords:** PSA, localized prostate cancer, phospholipids, triglycerides, tricarboxylic acid cycle, 2-hydroxyglutarate

## Abstract

**First-line treatment for localized prostate cancer (PCa) includes radical prostatectomy (RP) for high-risk disease.** However, in many cases, patients experience biochemical recurrence (BCR), heralded by rising prostate specific antigen (PSA) levels in the serum. Our goal was to identify metabolic pathways that are disrupted in BCR to determine potential targets of therapy. We conducted metabolomic analysis in prostate tissue from the tumors of 74 patients who underwent prostatectomy as treatment for localized PCa and correlated levels of metabolites with clinical and non-clinical factors. Cholesterol and triglycerides were upregulated in Hispanic vs. non-Hispanic and in obese vs. non-obese individuals, respectively. Both lipids and non-lipids were altered with increasing Gleason grades and clinical stages. High post-RP PSA (>0.1 ng/mL) indicated recurrence (*p* = 0.0094) and correlated with alterations in 141 metabolites including 114 lipids and 26 non-lipid molecules. The largest increase with high post-RP PSA was in 2-hydroxyglutaric acid (2-HG), a product of the tricarboxylic acid (TCA) cycle, that had previously been established as an oncometabolite in other cancers. 2-HG was highly selective and specific for high post-RP PSA (AUC = 0.8526; *p* = 0.0002) while Kaplan–Meier curves indicated that among patients who recurred, high 2-HG in the tumor reduced time-to-recurrence from 84 months (for those with low 2-HG) to 38 months (for those with high 2-HG). The addition of D2HG, an enantiomer of 2-HG, increased the growth rate of LNCaP and C4 cells, and also increased Akt and ERK phosphorylation. 2-HG is upregulated in PCa tumors from patients who experience high post-RP PSA indicative of recurrence. Future studies may target this metabolite to prevent recurrent disease.

## 1. Introduction

Localized PCa is typically diagnosed following an increase in the serum levels of the protease prostate-specific antigen (PSA) [1]. The standard of care for this disease ranges from active surveillance to radiation therapy (RT) or radical prostatectomy (RP) [2]; however, many patients experience biochemical recurrence (BCR) [3] following these treatments. Salvage therapies after definitive local treatment typically include pelvic RT often in combination with androgen deprivation therapy (ADT), which includes gonadotropin-releasing hormone (GnRH) agonists, alone [4] or together with the androgen synthesis inhibitor abiraterone acetate [5] or the androgen receptor (AR) inhibitors enzalutamide [6], apalutamide [7] or darolutamide [8]. Although most patients initially respond to ADT, they eventually develop castration resistant PCa (CRPC) [9]. Rising PSA values beyond 0.1 ng/mL following RP is the most common indicator of AR-dependent BCR and may involve metastatic disease [10]. While the 5-year overall survival for localized PCa is ~100%, that for distant metastasis is ~35%, but the signaling pathways leading to BCR are not completely known [11]. The overall goal of the current project is to identify factors involved in high post-RP PSA induction, indicative of BCR, via analysis of biochemical pathways and molecular mechanisms of PCa and ensuring that these changes affect ERK and Akt phosphorylation similarly.

BCR-related cellular alterations have been pursued by various laboratories, but most involve the identification of genes differentially expressed in patients undergoing BCR vs. those who did not [12,13]. However, activation of proteins encoded by these genes and expression of metabolites as a result of these protein activities are often ignored. Metabolomics identifies the products of these pathways and may be more relevant to clinical outcome due to its presence in the tumor microenvironment (TME) [14].

Most metabolomics studies of BCR have been conducted in liquid biopsies. A 2019 study collected blood samples prior to RP from 1766 patients with localized PCa and were followed up after surgery for a median time of 73.2 months [15]. Of these patients, 524 (29.67%) experienced BCR and 75 (4.25%) developed metastatic disease. Only steroids were measured in plasma using mass spectrometry (MS), and a one-unit increment in log-transformed androstenediol (A5diol) and dehydroepiandrosterone-sulfate (DHEA-S) levels were linked to disease-free survival (DFS) [15]. In another study, pre-surgical serum samples collected from a longitudinal PCa patient cohort (N = 382), 72 (19%) experienced a BCR event with a median follow-up time of 6.9 years [16]. This study, using global proteomics and lipidomics, indicated that two proteins, Tenascin C (TNC) and Apolipoprotein A1V (Apo-A1V), one metabolite, 1-Methyladenosine (1-MA), and one phospholipid, phosphatidic acid (PA) 18:0–22:0, showed a cumulative predictive performance of AUC = 0.78 [OR (95% CI) = 6.56 (2.98–14.40), *p* < 0.05] in differentiating patients with and without BCR events [16].

As serum and plasma analyses do not reflect the conditions at the TME, few studies have pursued metabolomics analysis of RP samples. A 2010 study on ex vivo tissue from 16 men with BCR and 16 age- and Gleason-score-matched individuals who experienced no BCR within 48 months after surgery, identified, using nuclear magnetic resonance (NMR) spectroscopy-based metabolomic analysis, four pathology-related principal components that predicted recurrence with an accuracy of 78% [17]. A later study with matched malignant and nonmalignant RP samples from 95 PCa patients using gas chromatography/liquid chromatography–mass spectrometry (GC/LC-MS) showed that levels of aminoadipic acid, gluconic acid and maltotriose were associated with BCR [18]. However, since none of these metabolites have been identified as an oncometabolite, we investigated frozen prostatectomy tissues in search of oncometabolites associated with BCR.

To attain this goal, we assessed frozen RP specimens from a cohort of 74 patients, 32.2% of whom later experienced BCR. These specimens were used to conduct untargeted metabolomic, lipidomic and biogenic amine analysis. We demonstrate dysregulation of components of the tricarboxylic acid (TCA) cycle in prostate tumors from patients who experience elevated post-RP PSA, which we used as a measure of BCR. Outside of the TCA cycle, our analysis showed that fatty acids such as triglycerides are upregulated while phospholipids are downregulated. While TCA cycle dysregulation and phospholipid reduction has previously been reported in PCa compared to normal prostate, this is the first report showing 2-HG elevation in BCR following RP. These results are significant, as 2-HG has previously been identified as an oncometabolite in various tissues [19].

## 2. Results

### 2.1. Clinicopathological Characteristics of Radical Prostatectomy Patients for This Study

Clinicopathological characteristics of 62 of the 74 patients with complete metabolomic data are presented in Table 1. The patients were overwhelmingly White (64.5%), and non-Hispanic (72.6%). The majority were never smokers (51.6%) or former smokers (41.9%). The median age of patients was 63.5 years (44–76). The median PSA at the time of diagnosis was 7.1 (CI:1.0 to 61.0 ng/mL). The majority had intermediate-grade tumors (Gleason score 7) (58.1%), while many had high-grade tumors (Gleason 8, 9) (33.9%), and only two (3.2%) had low-grade cancer (Gleason 6). Similarly, half the patients (50%) had T2 disease, while 41.9% had T3. One (1) patient (1.6%) had T4 disease at the time of diagnosis, manifested as bladder cancer, and had undergone cystoprostatectomy for muscle invasive bladder cancer. The median body mass index (BMI) for the patients at the time of surgery, calculated based on body weight and height, was 28.0—ranging from 20.0 to 61.2. After surgery, the majority of patients were negative for surgical margins (61.3%), but 32.2% were margin positive, possibly related to increased post-prostatectomy PSA in some patients. BCR is defined as a rise in PSA following RP or RT treatment, indicating tumor recurrence. A detectable postoperative PSA of >0.1 ng/mL within 4 to 8 weeks of surgery is a widely recognized predictor of BCR [20]. While 58% maintained post-prostatectomy PSA ≤ 0.1 ng/mL, 21% experienced elevated PSA levels post-prostatectomy. Median follow-up was 33.5 months. Three years after surgery, 32.2% of patients were declared to experience biochemical or clinical recurrence while 61.3% did not.

### 2.2. Ethnicity and BMI Determine the Expression of Differentially Expressed Lipids in Prostatectomy-Derived Tissues from Patients with PCa

Untargeted metabolomic analysis was conducted in the 74 aforementioned frozen RP PCa samples and normalized to referenced standards. Analysis was conducted on 3051 metabolic compounds—518 primary metabolites (154 known), 1689 lipids (582 known) and 844 biogenic amines (146 known)—which were subsequently analyzed and statistically correlated to age, BMI, smoking, race, ethnicity, tumor stage, Gleason score, PSA at diagnosis, post-RP PSA and PSA recurrence. Those with a Benjamini–Hochberg false discovery rate adjusted *p*-value < 0.05 were considered significant.

**Figure 1:**
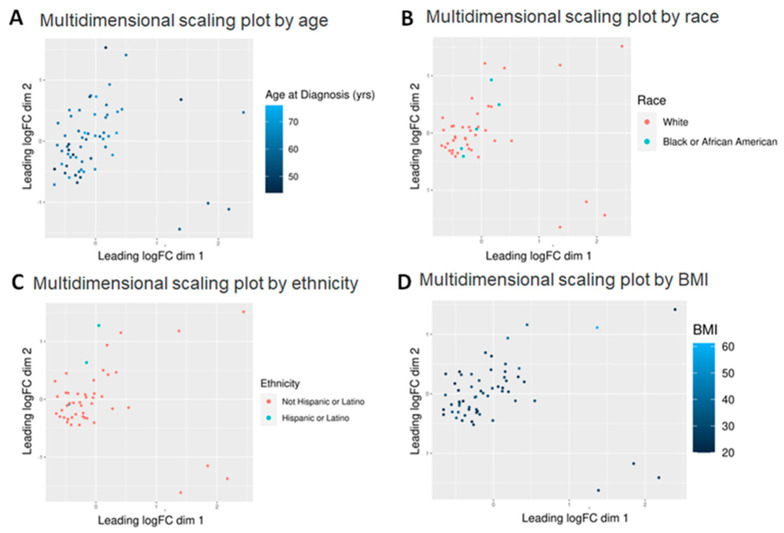
**Multidimensional scaling (MDS) plots by various factors characterizing the patients.** Graphical representation of relationships by abundance and significance of 882 known metabolites by (**A**) age (continuous variable—44–76), (**B**) race (White vs. Black), (**C**) ethnicity (Hispanic vs. non-Hispanic) and (**D**) BMI (continuous variable—20–61). Dimension reduction via MDS was achieved by taking the original set of samples and calculating dissimilarity (distance) measures for each pairwise comparison of samples. The samples were then represented graphically in two dimensions such that the distance between points on the plot approximates their multivariate dissimilarity closely.

We first considered the non-prostate specific factors (age, BMI, race, ethnicity, smoking). Of the five factors considered, age and BMI were continuous variables whereas race and ethnicity were discrete (Figure 1). Of 882 known metabolites, 48 showed some variation by age (*p* < 0.05), while 31 showed variations with respect to race (*p* < 0.05), but neither age nor race appeared to significantly affect the expression of the metabolites since adjusted *p*-value was >0.05 in all of them. Perhaps this was to be expected as the age of almost all the patients were within a 30-year range (5 [8.1%] in their 40s, 14 [22.6%] in their 50s, 34 [54.8%] in their 60s and 7 [11.3%] in their 70s—with 2 [3.2%] missing) (Figure 1A). Race may not have shown up as a factor perhaps since only five African American patients were featured in this group—perhaps a larger number would have shown some significant differences (Figure 1B). However, some recent papers have shown that the incidence of PCa is not all that different between the races when patients of similar socioeconomic and educational backgrounds were considered [21]. Hence it is not surprising that race alone did not show a significant change in metabolite expression.

**Figure 2:**
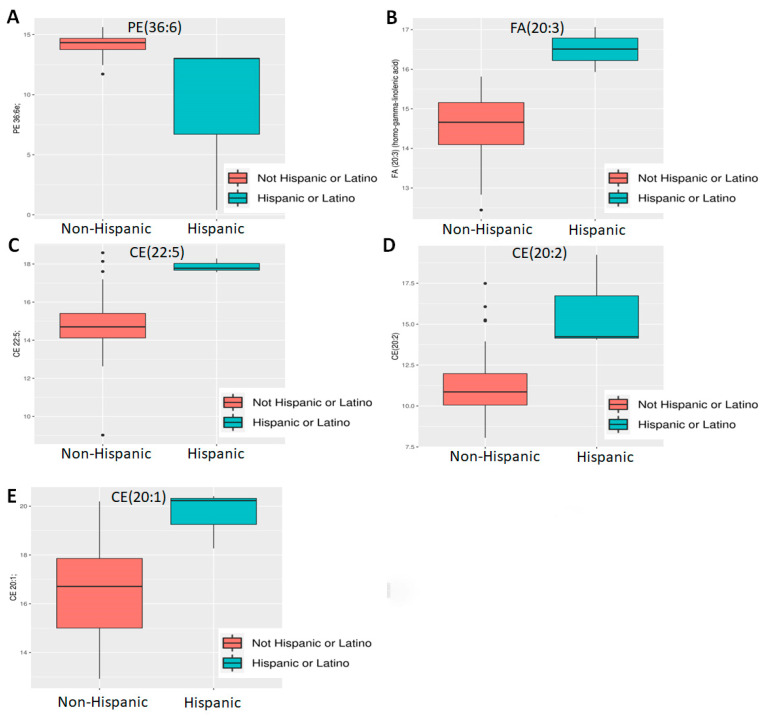
**Differential abundance analysis by ethnicity**. Five lipids—((**A**) PE 36:6e; (**B**) FA 20:3 [DGLA]; (**C**) CE 22:5, (**D**) CE 20:2; (**E**) CE 20:1) were found to be differentially expressed between Hispanics and non-Hispanics.

**Figure 3:**
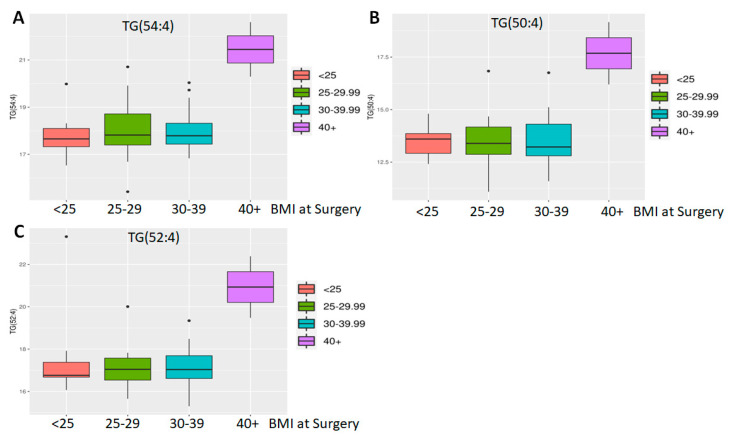
**Differential abundance analysis by BMI.** Three triglycerides ((**A**) TG 50:4; (**B**) TG 52:4; (**C**) TG 54:4) were found to be differentially expressed in patients with a very high BMI.

However, even with these limitations, ethnicity and BMI showed a significant difference. Of 882 known metabolites investigated, comparison of Hispanic (n = 3) vs. non-Hispanic (n = 45) tumors showed that 86 metabolites had *p* < 0.05 and of the latter, five also showed an adjusted *p* < 0.05 (Figure 1C). All are lipids, including three cholesteryl esters (CE 20:1; CE 20:2; CE 22:5), one (1) phosphatidylethanolamine (PE 36:6e) and one (1) essential fatty acid—FA (20:3) (dihomo-gamma-linolenic acid, DGLA). Of these, PE 36:6e levels declined while CE and DGLA levels increased in Hispanics compared to non-Hispanics (Figure 2). When BMI was analyzed, 80 metabolites were found to have *p* < 0.05, but of these, only three (3) were significant (adj *p* < 0.05) (Figure 1D). All three are triglycerides (TG 50:4; TG 52:4; TG 54:4), and these were elevated only in patients with extremely high BMI (40+) (n = 3) compared to the rest (n = 56) (Figure 3). Thus, we conclude that lipids were greatly affected by both ethnicity and BMI.

Smoking did not correlate with any significant difference in any metabolites Comparison of prostate tumor tissue from never smokers (n = 32) to former smokers (n = 26) showed that 57 metabolites exhibited *p* < 0.05 but adjusted *p*-value > 0.05. This could be because once the patient quits smoking metabolite composition is restored. There were too few current smokers (n = 2) (Table 1) to conduct any analysis with these patients in comparison to former or never smokers.

### 2.3. Decreased Lipid and Non-Lipid Metabolite Expression with Increased Gleason Score and Tumor Stage

**Figure 4:**
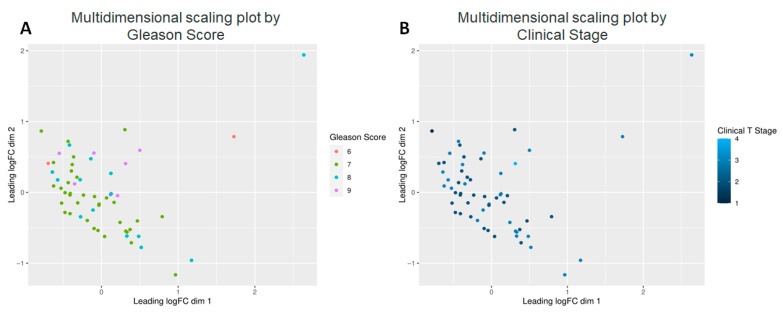
**Multidimensional scaling plots by clinical factors characterizing the patients.** Graphical representation of relationships by abundance and significance of 882 known metabolites by (**A**) Gleason score, and (**B**) Clinical Stage.

Next, we investigated the effect of PCa specific factors on the expression of the 882 known metabolites. Gleason score, a PCa specific grading system, and tumor stage were both evaluated (Figure 4). Comparison of Gleason scores 6, 7 (Low intermediate, n = 38) vs. 8, 9 (High, n = 21) yielded 120 metabolites at *p* < 0.05 and of these 12 were highly significant (adj. *p* < 0.05) (Table 2). This included three primary metabolites—phosphoethanolamine, asparagine and aconitic acid; lipids, which included a ceramide (Cer d41:1), two PCs (38:2e, p-36:1) and a lysophosphatidylcholine (LPC 18:1); as well as secondary metabolites, including erucamide, β-nicotinamide adenine dinucleotide (NAD+), hydroxychloroquine, Lauroyl-L-carnitine and deferiprone.

**Figure 5:**
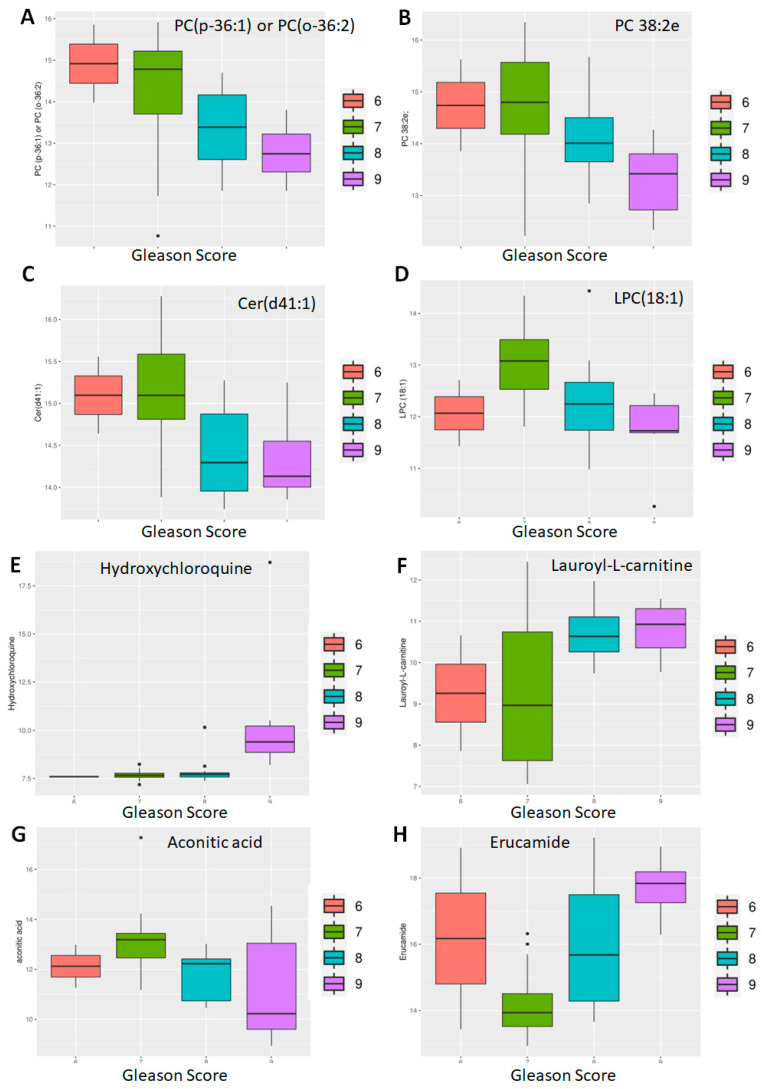
**Differential abundance by Gleason score: lipids and non-lipids.** Eight of twelve metabolites differentially expressed between patients with well-differentiated (Gleason 6), moderately differentiated (Gleason 7) and poorly differentiated (Gleason 8, 9) tumors are shown here. Of these, four are lipids (**A**–**D**) and four non-lipids (**E**–**H**).

The differential expression of some of these metabolites is shown in Figure 5. Among the lipids, there was a clear trend of lower lipid levels in case of higher Gleason score [PC (p-36:1) or PC (o-36:2), PC 38:2e, Cer (d41:1)] (Figure 5A–C), with the exception being LPC (18:1) (Figure 5D). With the first three, it appears that well-differentiated (Gleason 6) and moderately differentiated (Gleason 7) tumors expressed higher levels of the lipid compared to the poorly differentiated (Gleason 8, 9) ones.

However, metabolites like hydroxychloroquine were significantly upregulated in Gleason 9 tumors compared to the rest (Figure 5E). Similarly, there was a clear separation between well—and moderately differentiated (Gleason 6, 7) tumors compared to poorly differentiated (Gleason 8, 9) tumors with respect to lauroyl-L-carnitine (Figure 5F) and asparagine (Figure 5G) with the poorly differentiated ones expressing higher levels. In contrast, erucamide showed a gradual increase in Gleason score (Figure 5H).

**Figure 6:**
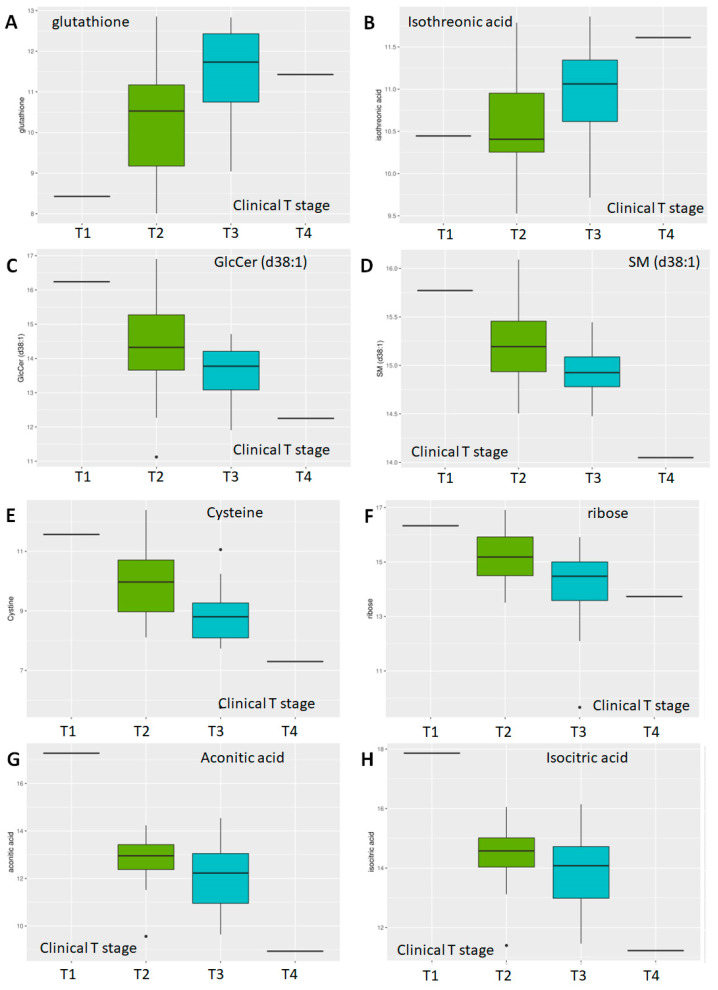
**Differential abundance by clinical T-stage.** Multidimensional scaling differential abundance analysis by clinical stage—T1/T2/T3/T4 for (**A**) the anti-oxidant glutathione, (**B**) isothreonic acid, a metabolite of Vitamin C, (**C**) the glycosphingolipid glucosylceramide d38:1, (**D**) the sphingolipid Sphingomyelin d38:1, (**E**) Cysteine, a non-essential amino acid, (**F**) ribose, a simple sugar, and two components of the TCA cycle (**G**) Aconitic Acid and (**H**) Isocitric Acid.

With respect to tumor stage, of available 882 metabolites, 153 showed *p* < 0.05; of the latter, 14 showed adj. *p* < 0.05 when stages T1 and T2 (n = 32) were compared to stages T3 and T4 (n = 27) (Table 3). Of these, four are lipids—a sphingomyelin (SM d38:1), two LPC (16:0, 18:1), and a glucosylceramide (GlcCer d38:1), and a secondary amine—putrescine. The majority, however, were primary amines—including cystine, glutathione, ribose, arabinose, phosphoethanolamine, dehydroascorbic acid, isothreonic acid, isocitric acid, and aconitic acid. Of these, two metabolites increased with increasing clinical stage-glutathione (Figure 6A) and isothreonic acid (Figure 6B), while the lipids, as with Gleason scores, also decreased with increasing tumor stage (Figure 6C,D). Overall, the majority of metabolites decreased with increased Gleason score and tumor stage, especially the lipids, with a few notable exceptions.

### 2.4. Differential Expression of Lipid and Non-Lipid Metabolites with Increasing Post-RP PSA

**Figure 7:**
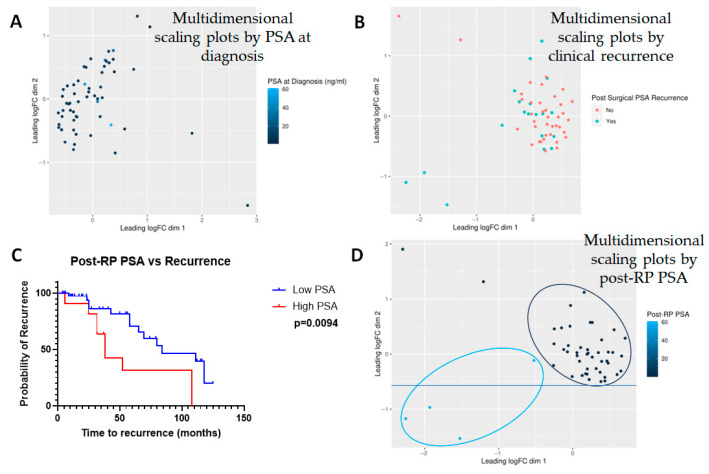
**Multidimensional scaling plots by PSA-related measures.** Multidimensional scaling plots by (**A**) PSA at diagnosis and (**B**) post-surgical PSA recurrence. Note that the latter includes not only post-surgical PSA levels but also a diagnosis of clinical recurrence based on imaging studies. Neither factor showed any significant correlation with any of the metabolites investigated (n = 882). However, (**C**) Kaplan–Meier curve showed that post-prostatectomy PSA heavily influenced recurrence and (**D**) hence, post-prostatectomy PSA was considered to be a surrogate for biochemical recurrence (BCR). Note that in this case, multidimensional scaling showed a clear separation between those with low vs. high PSA.

Finally, we tested the interaction between PSA and the expression of various metabolites. PSA at the time of diagnosis did not turn out to be a differentiating factor between the metabolites (Figure 7A). When recurrence was considered a discrete incidence (yes vs. no), there was no significant correlation with the expression of any particular metabolite (Figure 7B). However, when post-RP PSA was considered as a continuous variable, and patients were clustered as those with post-RP PSA ≤ 0.1 (n = 36) vs. those with post-RP PSA > 0.1 (n = 13), Kaplan–Meier analysis showed a clear separation between those groups with median time-to-recurrence for the low post-RP PSA group at 84 months, whereas that for high post-RP PSA was 38 months (*p* = 0.0094) (Figure 7C). The hazard ratio for post-RP PSA > 0.1, an accepted predictor of BCR [20], was 3.924 (1.398–11.02). There was strong correlation between the expression of the metabolites and post-prostatectomy PSA levels (Figure 7D). While it is not an indication of causality, it may be noted that the low (n = 36) vs. high (n = 13) post-RP PSA patients clustered differentially—and there was a clear separation between the two groups. In addition, post-RP PSA correlated strongly with the clinical stage (R = 0.2795, *p* = 0.038).

**Figure 8:**
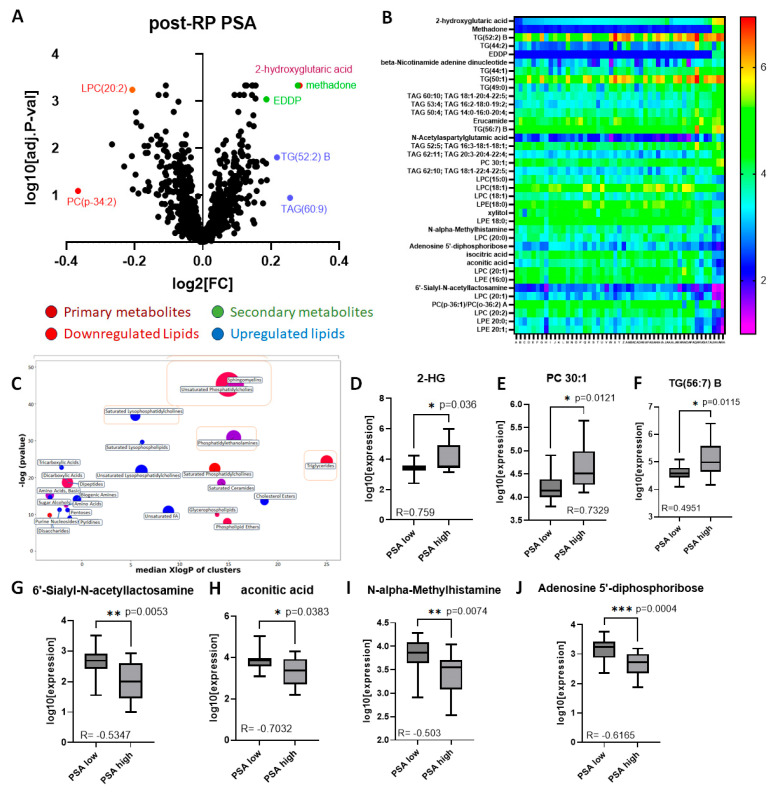
**Analysis of significant metabolites by postoperative PSA.** (**A**) Volcano plot of post-BCR PSA fold-change (LOG_2_FC) vs. significance (-LOG_10_ adjusted *p*-value), each point of which represents the median of three technical replicates. (**B**) Heatmap of top metabolites with increasing post-RP PSA (left to right). Each point represents the median of three technical replicates. (**C**) ChemRICH similarity enrichment showing the most significantly impacted metabolites differentially expressed in high vs. low post-RP PSA (*p* < 0.05). (**D**–**F**) Selected metabolites upregulated in high post-RP PSA patients; (**G**–**J**) selected metabolites downregulated in high post-RP PSA patients (Pearson correlation coefficients embedded in each figure).

There were 294 differentially expressed metabolites with *p* < 0.05; of these 141 were significant (adj. *p* < 0.05), including 12 primary metabolites, 14 secondary compounds and 115 lipids. Of these, 5 primary and 7 secondary metabolites and 67 lipids were upregulated while 7 primary and 7 secondary metabolites and 48 lipids were downregulated. A volcano plot was developed to identify the most important compounds (Figure 8A). The most significantly up- or downregulated among these were chosen for further analysis (38 identified in Table 4). A heatmap of these metabolites was created to display highlighted metabolic variation between samples designated as high or low for post-RP PSA (Figure 8B). Clear differential expressions of metabolites were shown with the upregulation of 2-HG in high post-RP PSA patient samples vs. low post-RP PSA samples.

ChemRICH analysis allowed for clustering based on chemical structure similarity and ontology mapping followed by enrichment statistics to yield study-specific and non-overlapping targets of self-contained size, and it confirmed many of the associations suspected (Figure 8C). Of these several metabolites correlated strongly with post-RP PSA levels (Pearson correlation coefficient > 0.3, *p* < 0.05) (Table 5). The ones showing the highest fold-change [upregulated (n = 3), downregulated (n = 4) are depicted as bar graphs (Figure 8D–J). Lipids comprised the majority of the metabolites differentially upregulated in prostate tissue in patients with high post-RP PSA; however, the one with the highest correlation with post-RP PSA was a non-lipid (2-hydroxyglutarate, 2-HG) (Figure 8D), while several non-lipids were downregulated (Figure 8G–J).

### 2.5. Identification of Significant Metabolites with Most Discriminatory Power to Distinguish Between Low vs. High Post-RP PSA

**Figure 9:**
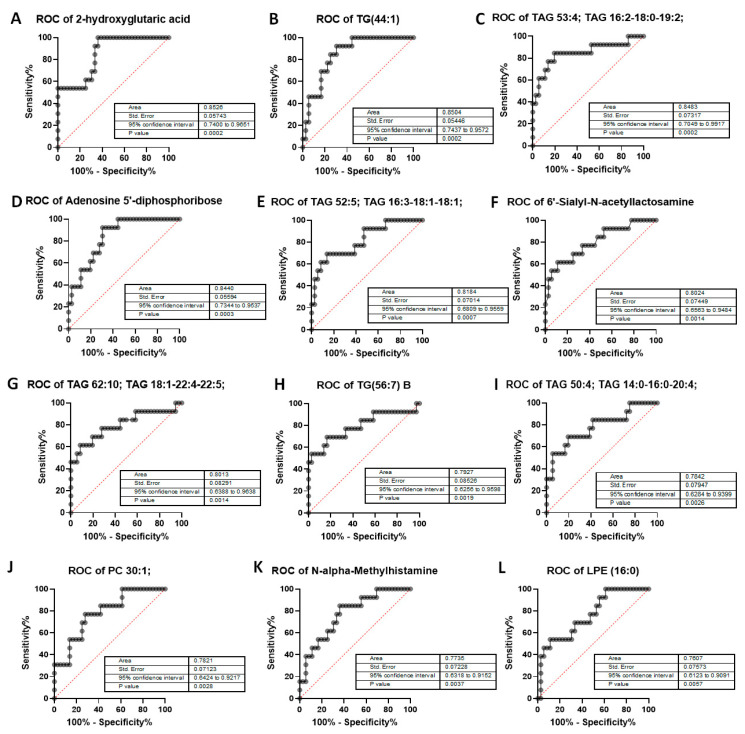
**Area Under the Curve (AUC > 0.75) of metabolites in post-RP PSA raw data analysis.** AUC has been calculated for—(**A**) 2-hydroxyglutaric acid, (**B**) triglyceride TG 44:1, (**C**) triacylglycerol 53:4, (**D**) Adenosine-5’-diphosphoribose, a product of NAD+ metabolism, (**E**) triacylglycerol 52:5, (**F**) 6′-Sialyl-N-acetyllactosamine, (**G**) triacylglycerol 62:10, (**H**) triglyceride 56:7, (**I**) triacylglycerol 50:4, (**J**) phosphatidylcholine 30:1, (**K**) N-alpha-Methylhistamine, (**L**) lysophosphatidylethanolamine (LPE) 16:0. Data inputs: controls (low post-RP PSA ≤ 0.1) vs. patients (high Post-RP PSA > 0.1). *p*-values are highly significant with no *p* > 0.005, representing 99.5% significance and greater. AUC > 0.75 cutoff to ensure most-significant metabolites as related to post-RP PSA increase or BCR.

We next tested the selectivity and specificity of the metabolites to determine whether they could be good predictors of high post-RP PSA. The Area under the Receiver Operator Characteristic (ROC) Curve (AUC) was calculated for all metabolites in Table 5. However, of these, only 12 metabolites showed an AUC > 0.75, which is deemed to be sufficiently discriminatory. Individual ROC curves for the 12 metabolites are shown in Figure 9. The results show that of all the metabolites tested, 2-HG, considered an oncometabolite [22], was the most discriminatory (AUC > 0.85, *p* = 0.0002). Additionally, 2-HG is part of the TCA cycle, and is expressed in all cells, including prostate cells. It was also the most correlated with post-RP PSA; hence we focused on this pathway for further analysis.

2-HG is a byproduct of α-ketoglutarate (α-KG), an intermediate product of the TCA cycle; it is generated by a reduction in the ketone group of α-KG to a hydroxyl group [22]. It can be found in two enantiomeric forms, L-2-hydroxyglutarate (L2HG) (also known as S-2HG) and D-2-hydroxyglutarate (D2HG) (also known as R-2HG) [23]. D2HG is increased in tumors harboring mutations in isocitrate dehydrogenase (IDH1), while L2HG accumulates in hypoxic and acidic conditions via activation of malate dehydrogenase 1 and 2 (MDH1 and MDH2) and lactate dehydrogenase A (LDHA). K-fold cross-validation of the AUC for 2-HG confirmed results from Figure 9A (Supplementary Figure S1).

### 2.6. Validation of 2-Hydroxyglutarate as a Regulator of PCa Proliferation In Vitro

**Figure 10:**
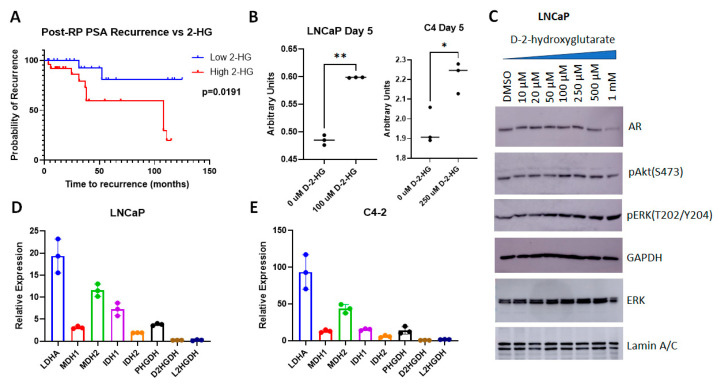
**Analysis of the role of 2-HG in PCa cells.** (**A**) Kaplan–Meier curve showing increased time to recurrence in patients with lower 2-HG. (**B**) MTT assay showing increased viability of LNCaP and C4 cells with D2HG (solubilized in DMSO) vs. vehicle (DMSO) treatment (n = 3 biological replicates per arm). ** *p* = 0.002, * *p* = 0.0205. (**C**) Immunoblots showing increased ERK phosphorylation following treatment with increasing levels of D2HG. (**D**,**E**) qPCR of untreated (**D**) LNCaP and (**E**) C4 cells display dehydrogenase activity, relative to GAPDH.

The above results indicate that higher 2-HG correlates with an increase in viability in the tumor cells, which may manifest itself in a poorer outcome. To test this hypothesis, we analyzed the relationship between 2-HG levels and the time to recurrence, represented by an increase in post-RP PSA (Figure 10A). We show that patients with high 2-HG levels (above median) were more likely to recur or recurred sooner compared to those with low 2-HG (at or below median) (*p* = 0.0191). The log-rank hazard ratio for recurrence in case of high 2-HG was calculated to be 4.802 (1.615–14.28).

Next, we investigated whether an increase in D2HG, which has previously been reported to be a growth promoter in PCa cell lines [24], affected cellular functions. As models we used hormone sensitive LNCaP cells, which represent recurrent tumors, as they were initially isolated from lymph node metastases, and its hormone insensitive C4 subline. D2HG was used to treat LNCaP and C4 cells for 5 days—after which cell metabolism/viability was evaluated via MTT assay (3-(4,5-dimethylthiazol-2-yl)-2,5-diphenyltetrazolium bromide colorimetric assay) (Figure 10B). Dose response curves showing the effect of increasing levels of D2HG are shown in Supplementary Figure S2. In LNCaP cells, 100 µM of D2HG treatment correlated with a substantial increase in viability (*p* = 0.002), as did 250 µM D2HG in C4 cells (*p* = 0.0205). Previous studies showed that 40 µM D2HG was required to have an effect in PC-3 cells, whereas in C4-2B cells, a 5-fold increase in concentration failed to have an effect [24]. The authors of that paper conclude that higher levels of AR inhibited the ability of D2HG to affect cell function. As C4 cells have higher AR transcriptional activity compared to LNCaP [25], whereas PC3 cells are AR null [26], these results suggest that the efficacy of D2HG inversely correlates with AR transcriptional activity.

To determine the effect of D2HG on downstream targets, we tested whether an increase in D2HG resulted in changes in cell signaling. D2HG did not have an effect on AR levels in LNCaP cells except at very high doses, but there was an increase in Akt phosphorylation at Ser 473 with ≥100 µM D2HG (Figure 10C), supporting our previous assertions regarding the importance of phospho-Akt (Ser 473) in post-RP PSA recurrence [27,28]. The change in ERK phosphorylation was even more pronounced, starting at ≥50 µM, likely causing an increase in viability and cell growth (Figure 10C). Individual blots and band quantitation are shown in Supplementary Figure S3. Thus, an increase in D2HG as seen in cells with elevated post-RP PSA can herald an increase in proliferation and viability in PCa patients.

Finally, we investigated the basis of the increase in D2HG seen in post-RP PSA. As mentioned earlier—D2HG correlates with the expression of mutant IDH1 and can also be affected by activation of malate dehydrogenase 1 and 2 (MDH1 and MDH2) and lactate dehydrogenase A (LDHA). To identify the relative expression of the genes involved in this pathway in LNCaP and C4 cells, we examined their expression by qPCR. LDHA was the most highly expressed gene in both cell lines, followed by MDH2, whereas the relative expression of most other genes varied between the two cell lines (Figure 10D,E). IDH1 was of higher relative expression in LNCaP cells whereas PHGDH was elevated in C4 cells. Overall, the relative expression was higher in C4 cells compared to LNCaP.

## 3. Discussion

In this study, we investigate the effects of various factors on the expression of select metabolites in PCa samples from patients who had undergone RP. Significantly, ethnicity appeared to have a fairly significant effect on certain lipids in the prostate tissue, while differentially expressed triglycerides were seen in the prostate in patients with extreme BMI (≥40). These results confirmed the validity of the results—as it is well known that high BMI levels result in increased circulating triglycerides [29], and that various ethnic diets strongly affects both cholesterol and fatty acid levels [30,31].

PCa-specific factors including Gleason score and clinical stage also affected metabolism greatly. Both lipids (decreases in phospholipids (PC, LPC) and sphingolipids (ceramide) and non-lipids (increase in erucamide, hydroxychloroquine, asparagine and lauroyl-L-carnitine, while reducing aconitic acid) were associated with increased Gleason score. Similarly, we see a decrease in cystine, aconitic acid, ribose and isocitric acid with increasing clinical stage, along with an increase in glutathione and isothreonic acid; while again, sphingolipids (SM and GlcCer) were decreased. Thus, both phospholipids and sphingolipids were decreased in more aggressive cancers. Phospholipids and sphingolipids are the main components of cell membranes and maintain membrane structure, thereby assisting in cell signaling and communication [32]. Loss and/or dysregulation of these lipids are often seen in tumor progression [33,34].

PSA is an established marker of PCa progression [1]. Following prostatectomy, any PSA level above the minimal is considered to be a sign of recurrence [3]. Here we see that the expression of metabolites from the prostatectomy samples correlates with post-RP PSA levels and not with the clinical diagnosis, which comes after many months. Elevated post-RP PSA levels correlated with changes in metabolism that were small but significant. Among lipids, phospholipids (PCs, LPCs and LPEs) were mostly decreased (as seen with high Gleason and T-stage), a phenomenon associated with cancer cachexia [35]. On the other hand, TGs and TAGs were upregulated in high-grade tumors. Elevated serum TGs are often associated with increased risk of PCa recurrence [36]. These results are therefore consistent with previous reports.

In addition to lipids, several non-lipid metabolites belonging to select pathways—including the tricarboxylic acid (TCA) pathway (isocitric acid, aconitic acid, 2-HG) [37] and the related NAD+ pathway (Nicotinamide, β-Nicotinamide adenine (NAD+, NAAG and its precursor NAA) [38], Adenosine 5′-diphosphoribose (ADPR), generated by the hydrolysis of NAD+ [39])—were altered. Methadone, a synthetic opioid used to treat cancer pain [40], and its metabolite EDDP [41], were both upregulated in patients with high post-RP PSA. In contrast, guanosine, a neuroprotectant [42], and N-Acetylglucosamine, which prevents neurodegeneration [43], were downregulated. Note that these changes were relatively small—however, even small changes could reflect major alterations in the metabolism of the patients and in the prostate gland (see Table 6).

It is well known that recurrence is common in high-risk PCa characterized by high Gleason scores and an increased clinical stage [44], hence it is expected that metabolites would behave similarly in all three conditions. This includes erucamide, aconitic acid and NAD+. Many of these compounds play a significant role in AR transcriptional activity. Methadone and EDDP lower androgen levels and AR function [45]. The NAD-dependent catalytic function of sirtuin 1 (SIRT1) deacetylated AR lysine residues and inhibited coactivator-induced interactions between the AR amino and carboxyl termini, thereby blocking DHT-induced contact-independent PCa growth [46]. Fatty acids have also been shown to inhibit the binding of androgens to the AR [47].

The differential expression of these metabolites in low vs. high PSA patients was also demonstrated by ROC curves, and two of these showed AUC > 0.85—2-HG and TG (44:1). Of all these metabolites, 2-HG was the most highly discriminatory; hence we investigated this metabolite further in cell lines to understand its role in PCa better. In support of the findings in the patient cohort, we see that the more aggressive C4 cells expressed higher levels of 2-HG compared to less aggressive LNCaP. The 2-HG enantiomers D2HG is associated with a mutated form of IDH1, which promotes the formation of α-ketoglutarate (α-KG); D2HG is generated by a reduction in the ketone group of α-KG to a hydroxyl group and is formed in larger amounts in cells with mutated IDH1, which is considered to be oncogenic [48]. Treatment with D2HG induced increased viability in both LNCaP and C4 cells and also resulted in an increase in ERK phosphorylation. These results are in support of previous observations demonstrating the oncogenic properties correlating with the dysfunction of the TCA cycle [49]. In fact, levels of 2-HG corresponded to the time taken by the tumors to recur—the time to PSA recurrence was low in patients with low 2-HG, whereas in patients with high 2-HG, the time to recurrence was high. Despite the fact that the increase in 2-HG was small, it resulted in larger changes in related genes including IDH1 in the more aggressive cells, suggesting the power of this oncometabolite in tumor progression. A study of the literature suggests that zinc accumulates in the benign prostate, which inhibits citrate to-isocitrate conversion while malignant cells have lower intracellular zinc, which reactivates the TCA cycle [50]. This allows the utilization of citrate as an energy source, permitting tumor progression [51]. Taken together, the above illustrates the significant role of 2-HG in prostate cancer recurrence after prostatectomy.

## 4. Materials and Methods

**Specimen Retrieval.** Primary PCa tissues from frozen RP specimens of 74 patients extracted between 2010 and 2017 were obtained from the University of California Davis (UC Davis) Comprehensive Cancer Center Biorepository based on an IRB-approved protocol. As prostate cancer is a heterogenous disease, adjoining cores from the same patient, let alone distally situated cores of a patient’s prostate, could have very different Gleason scores, which, our data show, would have very different effects on metabolite expression. Hence, we have attempted to use samples mainly from the section of the tumor with the highest Gleason grade. Complete metabolomic data were available from 62 patients. Immediately following processing of the specimens, they are snap-frozen in liquid nitrogen, then transferred to and temporarily stored at −80 °C for 1–4 weeks, after which all specimens are transferred frozen and on dry ice to long-term storage at −150 °C. They remain at −150 °C until a request to retrieve them is provided to the biorepository. At the time of analysis, frozen RP specimens were gradually thawed to avoid tissue damage and then prepared for untargeted metabolomic analysis.

**Metabolomic Analyses.** Metabolomic analysis of frozen human post-RP tissue samples were submitted to the West Coast Metabolomics Center, UC Davis. The samples were prepared in triplicate and analyzed as described elsewhere [52]. Briefly, samples were spiked with a recovery standard cocktail, extracted in methanol, and dried under vacuum to remove organic solvents. The reproducibility of the extraction protocol was assessed by the recovery of the xenobiotic compounds spiked in every sample prior to extraction. The extracts were separated into three fractions and characterized using three independent platforms: 1° metabolites by GC-TOF MS yielded metabolites such as amino acids, sugars, and tricarboxylic acids. Complex lipids: (e.g., ceramides, cholesteryl esters, free fatty acids, phosphatidyl-cholines, -serines and -ethanolamines, lysophospholipids, sphingomyelins and triacylglycerols) were quantified from an acetonitrile/isopropanol gradient on a charged surface hybrid (CSH) LC column and positive and negative electrospray ionization Q-TOF MS. 2° and charged metabolites in the form of biogenic amines were measured by HILIC QTOF MS. Metabolites from pharmaceutical drugs or exposomes were also captured through untargeted analysis. Data from all three platforms were normalized to human plasma samples and integrated into one spreadsheet prior to biostatistical analysis.

**Cell Culture and Materials.** Human prostate cancer cell lines LNCaP clone FGC (CRL-1740) and C4 (CRL-3313) (ATCC, Manassas, VA, USA) were cultured in RPMI 1640 medium with 5% fetal bovine serum (Gemini Biologicals, West Sacramento, CA, USA) and 1% antibiotic–antimycotic solutions (Gibco/Thermo Fisher Scientific, Waltham, MA, USA). D-2-Hydroxyglutarate (D2HG) and MTT Assay Kits were purchased from Sigma Aldrich (St. Louis, MO, USA) as described below. Rabbit polyclonal antibodies for Androgen Receptor (CS-3202), Phospho-Akt (Ser473) Antibody #9271, Phospho-p44/42 MAPK (Erk1/2) (Thr202/Tyr204) Antibody #9101, p44/42 MAPK (Erk1/2) Antibody #9102, and rabbit monoclonal GAPDH (14C10) #2118 and Lamin A/C (CS-2032) were from Cell Signaling Technology (Beverly, MA, USA).

**Quantitation of D-2-hydroxyglutarate.** D-2-hydroxyglutarate (D2HG) was quantitated in 0, 5 and 45 µg of cell lysates and culture media using the enzyme (D)-2-hydroxyglutarate dehydrogenase coupled to reduction in NAD+ (Sigma-Aldrich, St. Louis, MO, USA, Cat # 53-84-9) as described by others [53]. Briefly, confluent cells were collected after removal of the medium and dissociated in CelLytic M (Sigma Aldrich, St. Louis, MO, USA, Cat # C2978), and 0, 5 and 45 µg of the lysate and the media were used for analysis. Lysates and media were deproteinized with perchloric acid solution and neutralized with potassium hydroxide (Deproteinizing sample preparation kit, Sigma-Aldrich, MAK341). Lysates and media were loaded on black 96-well plates (Thermo Fisher Scientific, Waltham, MA, USA, Cat # 137101), and 75 μL of D2HG Complete Reaction Mixture (Sigma Aldrich, St. Louis, MO, USA, Cat # MAK320) was added into each well. Plates were incubated at 37 °C for 60 min, protected and wrapped in aluminum foil. Fluorometric detection was carried out in triplicate with λ_excitation_ = 540 nm and λ_emission_  =  610  nm.

**3-[4,5-Dimethylthiazol-2yl]-2,5-diphenyl-tetrazolium bromide (MTT) assay.** MTT assay was performed as described by us elsewhere [54]. Cells were cultured in 24-well plates and treated as indicated. Following treatment, each well was incubated with 25 µL of 5 mg/mL 3-[4,5-dimethylthiazol-2yl]-2,5-diphenyl-tetrazoliuM bromide (MTT; Invitrogen, part of Thermo Fisher Scientific, Waltham, MA, USA, Cat # M6494) for 1 h in a 5% CO_2_ incubator at 37 °C, which converted the reactants to formazan in actively dividing cells. Proliferation rates were estimated by colorimetric assay reading formazan intensity in a plate reader at 562 nm.

**Immunoblotting.** Immunoblotting was performed as described elsewhere [54]. Briefly, cell lysate protein levels were quantitated via BCA assay (Pierce, part of Thermo Fisher Scientific, Waltham, MA, USA) and fractionated on 29:1 acrylamide-bis SDS–PAGE. Electrophoresis was performed at 150 V for 2 h using mini vertical electrophoresis cells (Mini-PROTEAN 3 Electrophoresis Cell, Bio-Rad, Hercules, CA, USA). The gels were electroblotted for 2 h at 200 mA using Mini Trans-Blot Electrophoretic Transfer Cell (Bio-Rad, Hercules, CA, USA) onto a 0.2 µM polyvinylidene difluoride membrane (Osmonics, Westborough, MA, USA). The blots were stained overnight with primary antibodies at 4 °C and detected by enhanced chemiluminescence (Thermo Fisher, Waltham, MA, USA) following incubation with a peroxidase-labeled secondary antibody (goat anti-rabbit IgG, Fc specific, Jackson ImmunoResearch, West Grove, PA, USA). Loading controls included housekeeping proteins GAPDH and Lamin A/C as noted in the legends to the figures used. Bands were quantitated and normalized to the loading controls as described.

**qPCR analysis.** qPCR was performed using the −2 delta-delta Ct method as described elsewhere [24]. Total cellular RNA was prepared utilizing the Qiagen RNeasy kit (Redwood City, CA, USA) based on the manufacturer’s protocol. RNA concentration and purity were assessed using a NanoDrop spectrophotometer (Thermo Scientific), and integrity was confirmed by agarose gel electrophoresis. cDNA was synthesized from 1 µg total RNA using the iScript cDNA Synthesis Kit from BioRad (Hercules, CA, USA) as per the manufacturer’s protocol. qPCR was performed using SYBR Green Master Mix, Applied Biosystems on a StepOnePlus Real-Time PCR System. Primer sequences were designed using Primer-BLAST (NCBI) and synthesized by Integrated DNA technologies (IDT, San Diego, CA, USA) (Table S1). Primer specificity was verified by melt curve analysis, which confirmed single, specific amplification products without primer-dimer formation. Amplicon sizes were also confirmed via agarose gel electrophoresis. Amplification efficiency for each primer pair was determined using a standard curve generated from serial dilutions of cDNA (5-point 1:5 dilution series). All primer pairs exhibited efficiencies between 90 and 110% and R^2^ values > 0.99. Relative gene expression was calculated using the 2^−ΔΔCt^ method. β-Actin was used as the internal control for normalization, and its stability was confirmed across experimental conditions showing low variation in Ct values. Real-time PCR was run using TaqMan Gene Expression Master Mix (Applied Biosystems, Grand Island, NY, USA) according to the manufacturer’s recommendations. β-Actin was used as the endogenous expression standard. Data was collected on an Applied Biosystems 7500 Fast machine and analyzed using the relative standard curve method. Each reaction was performed in triplicate, and each experiment was repeated at least three times independently.

**Statistical Analyses.** R version 4.2.1 (23 June 2022), R package limma v.3.52.2 [55,56] was used for biostatistical analyses of the raw data, incorporating variance weights from the limma function vooma [57]. Metabolomics data were log-transformed prior to analysis to minimize the impact of extreme values, then normalized using cyclic loess [58]. Focus was given to metabolites with adjusted *p*-values < 0.05 to prevent false positives when performing multiple statistical tests. No abundance filtering was conducted; however, the analysis included only metabolites with a BinBase name.

*Target Identification*: The BinBase database was used for target identification. Since untargeted assays were conducted, relative quantification rather than absolute quantification for the metabolites were used; however, the limit of detection (LOD) and the limit of quantitation (LOQ), which is different for different compounds, was previously reported to be 0.01–0.1 µg/mL [59]. *Pathway and enrichment analyses* were performed using ChemRICH software from the UC Davis West Coast Metabolomics core and through employment of the Homo sapiens Kyoto Encyclopedia of Genes and Genomes (KEGG) metabolic pathway database (FDR < 0.05, on pathways with 5 or more hits). Cited publications, Human Protein Atlas, and PubChem were used to determine metabolite potential function in PCa. Patients with missing data were not included in the analysis.

*Data Visualization*: The function plotMDS from limma was used to create the multidimensional scaling (MDS) plots, which by default use the Euclidean distance between the 500 metabolites differing the most between a pair of samples to create the distance matrix. The MDS plots are a distance-preserving two-dimensional display of the data, colored by patient characteristics. There is no statistical analysis that is associated with MDS plots, they are merely a visualization method. GraphPad PRISM 10 was utilized to create a heatmap, volcano plot and Kaplan–Meier and ROC curves.

## 5. Conclusions

This is the first time that enhanced activation of 2-HG is correlated with increased post-RP PSA and indicates a role for this pathway in PSA recurrence. Our results indicate a future role of IDH1 inhibitors, which selectively binds to and inhibits the mutated form of IDH1, thus reducing the production of 2-HG, in PCa treatment [60]. Examples of IDH1 inhibitors include ivosidenib (Tibsovo) and olutasidenib (Rezlidhia), two FDA-approved IDH1 inhibitors used in the treatment of relapsed or refractory AML with susceptible IDH1 mutations [60]. Vorasidenib is another IDH inhibitor targeting both IDH1 and IDH2 mutations, being investigated for low-grade gliomas [60]. While these drugs have not been investigated as of now in PCa, future studies may indicate their efficacy in the treatment of this elusive disease.

## Figures and Tables

**Table 1:** Patient characteristics.

		Overall
**Race**	White	40 (64.5%)
	Black or African American	5 (8.1%)
	Other	3 (4.8%)
	Not Reported	14 (22.6%)
**Ethnicity**	Hispanic or Latino	3 (4.8%)
	Not Hispanic or Latino	45 (72.6%)
	Not Reported/Unknown	14 (22.5%)
**Smoking Status**	Current	2 (3.2%)
	Former	26 (41.9%)
	Chewed Tobacco (Former)	1 (1.6%)
	Never	32 (51.6%)
	Missing	1 (1.6%)
**Age at Diagnosis (yrs)**	Mean (SD)	61.8 (7.33)
	Median [Min, Max]	63.5 [44.0, 76.0]
	Missing	2 (3.2%)
**PSA at Diagnosis (ng/mL)**	Mean (SD)	10.7 (10.9)
	Median [Min, Max]	7.13 [1.00, 61.0]
	Missing	5 (8.1%)
**Gleason Score**	Low-grade (Gleason 6)	2 (3.2%)
	Intermediate (Gleason 7)	36 (58.1%)
	High grade (Gleason 8, 9)	21 (33.9%)
	Missing	3 (4.8%)
**Clinical T Stage**	1	1 (1.6%)
	2	31 (50.0%)
	3	26 (41.9%)
	4	1 (1.6%)
	Missing	3 (4.8%)
**BMI at Surgery**	Mean (SD)	29.8 (6.72)
	Median [Min, Max]	28.0 [20.0, 61.2]
	Missing	3 (4.8%)
**Surgical Margin (Pos/Neg)**	Neg	38 (61.3%)
	Pos	20 (32.2%)
	Missing	4 (6.5%)
**Post RP PSA**	≤0.1	36 (58.0%)
	>0.1	13 (21.0%)
	Missing	13 (21.0%)
**PSA Recurrence**	No	38 (61.3%)
	Yes	20 (32.2%)
	Missing	4 (6.5%)

**Table 2:** Differentially expressed metabolites in low vs. high Gleason tumors.

Identifier/Ret.Index	BinBase Name/Annotation	Log_2_FC	Fold Change	*p*-Value	adj.P.Val
1.03_338.34	Erucamide	1.50	2.8284271	0.0000	0.0000
9.55_664.11	β-Nicotinamide adenine dinucleotide	1.23	2.3456699	0.0005	0.0375
6.40_336.18	Hydroxychloroquine	1.18	2.2657678	0.0000	0.0114
3.59_344.28	Lauroyl-L-carnitine	0.86	1.8150383	0.0002	0.0280
5.90_140.08	Deferiprone	0.75	1.6817928	0.0007	0.0478
603,912	Phosphoethanolamine	0.65	1.5691682	0.0005	0.0375
553,078	Asparagine	0.50	1.4142136	0.0000	0.0142
7.92_618.62_7.92_658.61	Cer(d41:1)	−0.43	0.7422618	0.0002	0.0280
1.65_580.36	LPC (18:1)	−0.50	0.7071068	0.0004	0.0375
6.73_800.66	PC 38:2e;	−0.66	0.6328783	0.0001	0.0229
586,815	Aconitic acid	−0.79	0.5783441	0.0005	0.0375
6.28_830.63_6.27_806.59	PC (p-36:1) or PC (o-36:2)	−0.81	0.5703819	0.0001	0.0148

**Table 3:** Differentially expressed metabolites in prostate tumors stages T1–T4.

Identifier/Ret.Index	BinBase Name/ Annotation	Log_2_FC	Fold Change	*p*-Value	adj.P.Val
761,296	glutathione	1.09	2.1287	0.0001	0.0181
603,912	phosphoethanolamine	0.88	1.8403	0.0005	0.0378
633,423	dehydroascorbic acid	0.65	1.5691	0.0008	0.0485
489,385	isothreonic acid	0.49	1.4044	0.0002	0.0215
6.22_793.60_6.22_817.64	SM (d38:1)	−0.33	0.7955	0.0003	0.0274
1.49_530.30_1.49_554.35	LPC (16:0)	−0.59	0.6643	0.0008	0.0495
1.65_580.36	LPC (18:1)	−0.63	0.6461	0.0007	0.0485
550,621	arabinose	−1.02	0.4931	0.0006	0.0421
616,323	isocitric acid	−1.02	0.4931	0.0002	0.0215
553,135	ribose	−1.02	0.4931	0.0001	0.0181
9.02_89.11_9.01_72.08	putrescine	−1.07	0.4763	0.0003	0.0310
6.48_790.59_6.47_814.64	GlcCer (d38:1)	−1.08	0.4730	0.0001	0.0181
586,815	aconitic acid	−1.19	0.4383	0.0001	0.0181
804,619	cystine	−1.44	0.3685	0.0000	0.0155

**Table 4:** Differentially expressed metabolites in prostate tumors with varying post-RP PSA.

Identifier/Ret.Index	BinBase Name/Annotation	FC	AveExpr	*p*-Value	adj.P.Val
506,306	2-hydroxyglutaric acid	1.22	11.87	3.47 × 10^−6^	0.0005
1.83_310.22	Methadone	1.21	8.47	1.15 × 10^−6^	0.0005
10.80_876.81_10.81_897.73	TG(52:2) B	1.16	18.35	0.0012837	0.0157
8.96_785.61_9.00_764.66_9.00_769.63	TG(44:2)	1.14	10.52	0.00768123	0.0476
1.72_278.19	EDDP	1.14	8.01	1.76 × 10^−5^	0.0009
9.55_664.11	β-Nicotinamide adenine dinucleotide	1.14	10.5	0.00208183	0.0211
9.42_771.65_9.45_766.69_9.45_787.62	TG(44:1)	1.13	13.2	0.00166434	0.0188
10.83_871.72_10.80_855.74_10.80_850.79	TG(50:1)	1.12	18.5	0.00270858	0.0244
10.94_843.74_10.93_838.79	TG(49:0)	1.12	12.87	0.00187129	0.0195
10.00_972.80	TAG 60:10; TAG 18:1–20:4–22:5;	1.11	12.53	3.42 × 10^−6^	0.0005
10.50_886.79	TAG 53:4; TAG 16:2–18:0–19:2;	1.11	11.78	1.28 × 10^−6^	0.0005
9.84_844.74	TAG 50:4; TAG 14:0–16:0–20:4;	1.11	13.34	1.59 × 10^−5^	0.0009
1.03_338.34	Erucamide	1.11	15.32	0.00279158	0.0244
10.17_927.75_10.19_922.79	TG(56:7) B	1.11	15.74	9.33 × 10^−5^	0.0034
8.01_305.10	N-Acetylaspartylglutamic acid	1.11	8.41	0.00173386	0.0193
9.87_870.75	TAG 52:5; TAG 16:3–18:1–18:1;	1.11	14.18	4.86 × 10^−5^	0.002
9.93_998.82	TAG 62:11; TAG 20:3–20:4–22:4;	1.11	11.15	2.91 × 10^−6^	0.0005
4.30_704.52	PC 30:1;	1.11	14.35	2.52 × 10^−6^	0.0005
10.37_1000.83	TAG 62:10; TAG 18:1–22:4–22:5;	1.1	11.29	1.11 × 10^−5^	0.0007
1.16_482.33	LPC(15:0)	0.92	12.34	0.00056155	0.0109
1.57_522.36	LPC(18:1)	0.92	16.86	0.0018648	0.0195
1.65_580.36	LPC (18:1)	0.92	12.44	0.0002319	0.0066
2.22_482.33	LPE (18:0)	0.92	16.39	0.00032532	0.0082
567,437	xylitol	0.92	13.98	0.00268863	0.0244
2.32_480.31	LPE 18:0;	0.91	15.32	0.0003659	0.0083
7.13_126.10_7.13_109.07	N-alpha-Methylhistamine	0.91	12.31	0.00118441	0.0152
3.00_552.40	LPC (20:0)	0.91	12.35	0.00018095	0.0054
9.66_560.07	Adenosine 5′-diphosphoribose	0.9	10.19	0.00022747	0.0066
616,323	Isocitric acid	0.89	13.86	0.00074461	0.0125
586,815	Aconitic acid	0.89	12.24	0.00081344	0.0126
2.43_608.39	LPC (20:1)	0.89	10.25	0.00043379	0.0089
1.51_452.28	LPE (16:0)	0.88	14.2	9.59 × 10^−5^	0.0034
9.45_675.24	6′-Sialyl-N-acetyllactosamine	0.87	8.49	7.11 × 10^−5^	0.0028
2.31_550.39	LPC (20:1)	0.87	14.15	4.01 × 10^−5^	0.0017
6.07_772.63	PC(p-36:1)/PC(o-36:2) A	0.87	14.39	0.00517676	0.0364
1.74_548.37	LPC (20:2)	0.87	13.15	5.78 × 10^−6^	0.0006
3.12_508.34	LPE 20:0	0.87	10.79	0.0010802	0.0148
2.47_506.32	LPE 20:1	0.85	11.19	0.00280752	0.0244

**Table 5:** Pearson correlation coefficient of differentially expressed metabolites in prostate tumors with varying post-RP PSA.

	Pearson R	95% Confidence Interval	R Squared	P (Two-Tailed)
PSAvs.2-hydroxyglutaric acid	0.759	0.6075 to 0.8573	0.5761	<0.0001
PSAvs.PC 30:1	0.7321	0.5678 to 0.8403	0.536	<0.0001
PSAvs.TAG 62:11; TAG 20:3–20:4–22:4	0.6747	0.4856 to 0.8034	0.4552	<0.0001
PSAvs.TAG 62:10; TAG 18:1–22:4–22:5	0.6457	0.4454 to 0.7845	0.4169	<0.0001
PSAvs.TAG 53:4; TAG 16:2–18:0–19:2	0.6165	0.4056 to 0.7651	0.3801	<0.0001
PSAvs.TAG 60:10; TAG 18:1–20:4–22:5	0.6151	0.4037 to 0.7641	0.3783	<0.0001
PSAvs.TAG 50:4; TAG 14:0–16:0–20:4	0.5712	0.3455 to 0.7345	0.3263	<0.0001
PSAvs.TAG 52:5; TAG 16:3–18:1–18:1	0.5133	0.2713 to 0.6943	0.2635	0.0002
PSAvs.TG(56:7) B	0.4951	0.2485 to 0.6814	0.2451	0.0003
PSAvs.TG(44:1)	0.3155	0.03763 to 0.5481	0.09953	0.0272
PSAvs.N-alpha-Methylhistamine	−0.503	−0.6870 to −0.2583	0.253	0.0002
PSAvs.6′-Sialyl-N-acetyllactosamine	−0.5347	−0.7093 to −0.2984	0.2859	<0.0001
PSAvs.LPE (16:0)	−0.6136	−0.7631 to −0.4018	0.3765	<0.0001
PSAvs.Adenosine 5′-diphosphoribose	−0.6165	−0.7650 to −0.4056	0.38	<0.0001
PSAvs.aconitic acid	−0.7032	−0.8219 to −0.5260	0.4944	<0.0001

**Table 6:** Classification of differentially expressed non-lipid metabolites in prostate tumors with elevated post-RP PSA.

Identifier/Ret.Index	BinBase Name/Annotation	Classification	Function
506,306	2-hydroxyglutaric acid	organic acid	
1.83_310.22	Methadone	synthetic opioid	pain relief, treat opioid use
1.72_278.19	EDDP	synthetic opioid	metabolite of methadone
9.55_664.11	β-Nicotinamide adenine (NAD+)	coenzyme	produce energy, DNA repair
1.03_338.34	Erucamide	fatty acid	a wax used in food packaging
8.01_305.10	N-Acetylaspartylglutamic acid	neuropeptide	peptide neurotransmitter
567,437	xylitol	sugar alcohol	sugar substitute
7.13_126.10_7.13_109.07	N-alpha-Methylhistamine	aralkylamino	migraine pain relief
9.66_560.07	Adenosine 5′-diphosphoribose	nucleotide sugar	NAD intermediary
616,323	Isocitric acid	organic acid	found in most fruit juices
586,815	Aconitic acid	organic acid	
9.45_675.24	6′-Sialyl-N-acetyllactosamine	oligosaccharide	

## Data Availability

All the authors have consented to the contents of the publication. No other consent is necessary as all the data presented have been generated by the authors. All available data have been presented in the manuscript. If additional details are requested, the authors may be contacted for further information. All materials generated for this paper, if available, can be shared with interested individuals against an MTA with the University of California, Davis.

## Supplementary Materials

The following supporting information can be downloaded at https://www.mdpi.com/article/10.3390/molecules30163316/s1; Supplementary Table S1. Primers used for qPCR, Supplementary Figure S1. Cross-validation of ROC Curve for 2-HG, Supplementary Figure S2. Dose response Curve for D2HG, Supplementary Figure S3. Quantitation of Bands in Western Blots.

## Abbreviations

The following abbreviations are used in this manuscript:

1-MA	1-Methyladenosine
2-HG	2-hydroxyglutaric acid
A5diol	Androstenediol
AC	Acetylcholine
adj P Val	Benjamini–Hochberg false discovery rate adjusted *p*-value
ADPR	Adenosine 5′-diphosphoribose
ADT	Androgen Deprivation Therapy
APO-A1V	Apolipoprotein A1V
AR	Androgen Receptor
AUC	Area under the ROC Curve
AveExpr	Average Expression
BCR	Biochemical recurrence
BLAST	Basic Local Alignment Search Tool
BMI	Body Mass Index
CE	Cholesteryl ester
Cer	Ceramide
ChemRICH	Chemical Similarity Enrichment Analysis
CI	Confidence Interval
CRPC	Castration resistant prostate cancer
CSH	Charged surface hybrid
D2HG	D-2-Hydroxyglutarate
DFS	Disease-free survival
DGLA	Dihomo-gamma-linolenic acid
DAG	Diacylglycerol
DHEA-S	Dehydroepiandrosterone-sulfate
ERK	Extracellular signal-regulated kinase
FA	Fatty acid
FC	Fold change
GC-TOF	Gas chromatography–time of flight
GlcCer	Glucosylceramide
GnRH	Gonadotropin Releasing Hormone
HILIC	Hydrophilic interactive liquid chromatography
IDH1	Isocitrate dehydrogenase
IRB	Internal Review Board
KEGG	Kyoto Encyclopedia of Genes and Genomes
L2HG	L-2-hydroxyglutarate
LC	Liquid chromatography
LDHA	Lactate dehydrogenase A
LNCaP	Lymph Node metastasis of Cancer of the Prostate
LPC	Lysophosphatidylcholine
LPE	Lysophosphatidylethanolamine
MAPK	Mitogen-activated protein kinase
MDH	Malate dehydrogenase
MDS	Multidimensional Scaling
MS	Mass spectroscopy
MTT	3-[4,5-Dimethylthiazol-2yl]-2,5-diphenyl-tetrazolium bromide
NAA	N-Acetylaspartate
NAAG	N-acetyl-aspartyl-glutamate
NAD+	β-Nicotinamide adenine
NMR	Nuclear magnetic resonance
OR	Odds ratio
PA	Phosphatidic acid
PCa	Prostate cancer
PC	Phosphatidylcholines
PE	Phosphatidylethanolamine
PLA2	Phospholipase A2
PSA	Prostate specific antigen
PTEN	Phosphatase and tensin homolog
qPCR	Quantitative polymerase chain reaction
QqQ-LC	Triple quadrupole liquid chromatography
Q-TOF	Quadrupole Time-of-Flight
ROC	Receiver Operator Characteristic
RP	Radical prostatectomy
RT	Radiation therapy
SM	Sphingomyelin
TAG	Triacylglycerol
TCA	Tricarboxylic acid
TG	Triglycerides
TME	Tumor Microenvironment
TNC	Tenascin C
α-KG	α-ketoglutarate
