# Supplementary material for: The Oncometabolite 2-Hydroxyglutarate Is Upregulated in Post-Prostatectomy PSA Recurrence of Prostate Cancer: A Metabolomic Analysis
Source: Molecules. 2025 Aug 8;30(16):3316. doi: 10.3390/molecules30163316 (PMC12388815; doi:10.3390/molecules30163316)

Supplementary Information

The Oncometabolite 2-Hydroxyglutarate is upregulated in Post-Prostatectomy PSA recurrence of Prostate Cancer: A Metabolomic Analysis

Dontrel W. Spencer Hairston[1,2], Shamira Sridharan-Weaver[2], Abheek Gandhi[3], Neelu Batra[1,4], Blythe P. Durbin-Johnson[5], Marc A. Dall'Era[2] and Paramita M. Ghosh[1,2,4,*]

Table of Contents:

Supplementary Table S1. Primers used for qPCR Page 1

Supplementary Figure S1. Cross-validation of ROC Curve for 2-HG Page 2

Supplementary Figure S2. Dose response Curve for D2HG Page 2

Supplementary Figure S3. Quantitation of Bands in Western Blots Page 3

Supplementary Table S1. Primers used for qPCR.

Gene	Direction	Sequence
LDHA	Forward	5'-AGCCCGATTCCGTTACCT-3'
LDHA	Reverse	5'-CACCAGCAACATTCATTCCA-3'
MDH1	Forward	5'-CTGTGACCACGTCAGGGACAT-3'
MDH1	Reverse	5'-TCTTGATTACAACAGGGAATGAGTAGAG-3'
MDH2	Forward	5'–CCTGTTCAACACCAATGCCA–3'
MDH2	Reverse	5'–GCCGAAGATTTTGTTGGGGT–3'
PHGDH	Forward	5'-CACGACAGGCTTGCTGAATGA-3'
PHGDH	Reverse	5'-CTTCCGTAAACACGTCCAGTG-3'
mIDH1-R132	Forward	5'-ACGGTCTTCAGAGAAGC-3'
mIDH1-R132	Reverse	5'-GGTGTAGATACCAAAAGATAAGAAT-3'
D2HGDH	Forward	5'-GGTGAGATCCTGTCTGCATTCG-3'
D2HGDH	Reverse	5'-TGGAGCCTGAAGTCTCGATGAG-3'
L2HGDH	Forward	5'-GTCATCGTTGGTGGCGGAATTG-3'
L2HGDH	Reverse	5'-CTGTTATGTCCAGTCTGGTGAAC-3'
GAPDH	Forward	5'-CGACCACTTTGTCAAGCTCA-3'
GAPDH	Reverse	5'-GGGTCTTACTCCTTGGAGGC-3'
Lamin A	Forward	5'-ATGAGGACCAGGTGGAGCAGTA-3'
Lamin A	Reverse	5'-ACCAGGTTGCTGTTCCTCTCAG-3'

Supplementary Figure S1. Cross-validation of ROC Curve for 2-HG.

The main text shows that the ROC curve for 2-HG is 0.8526 (0.74 – 0.9651), p=0.0002. To cross-validate the data, we used K-fold crossvalidation, where we divided the existing data arbitrarily into K-folds – one being used as a training set and the other as a validation set. The training set was used to compute the False Positive Rate (FPR) and True Positive Rate (TPR) at various probability thresholds and the AUC for the fold was calculated. This process was then repeated for the validation set. Note that the two folds had comparable AUCs (0.8796 for set 1; 0.8413 for set 2), and the mean of the two sets (0.8605) is <1% (0.92%) different compared to the one with the complete set, thus validating the results.

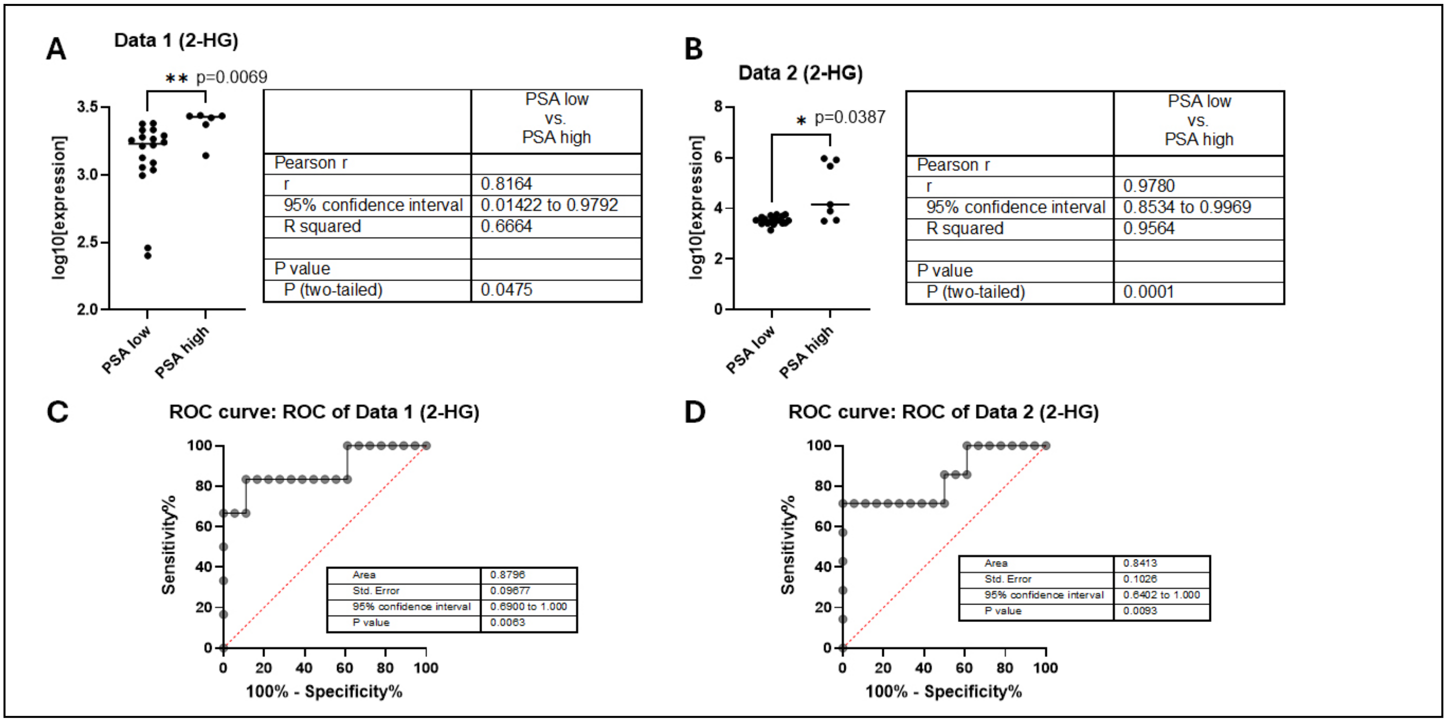

Supplementary Figure S2. Dose response Curve for D2HG.

Hormone sensitive LNCaP cells and its hormone resistant derivative C4, and an unrelated line 22Rv1, were treated with increasing doses of the oncometabolite D2HG. The cells were treated for 5 days and then collected and cell viability estimated by MTT assay. Results demonstrate a steady increase in cell viability at these doses.

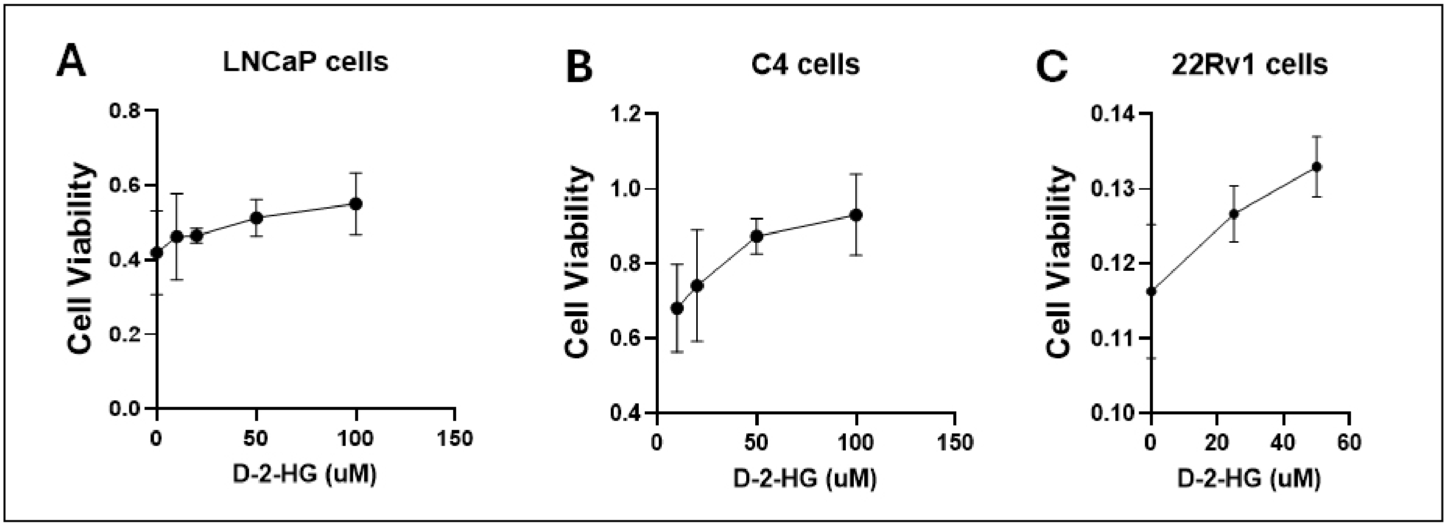

Supplementary Figure S3. Quantitation of Bands in Western Blots.

(A, D) The blots shown in Figure 10C are demonstrated below. **(B, C, E)** Bands were quantitated by Image J and normalized to the corresponding loading control (GAPDH for phospho-Akt and phospho-ERK and Lamin for ERK).

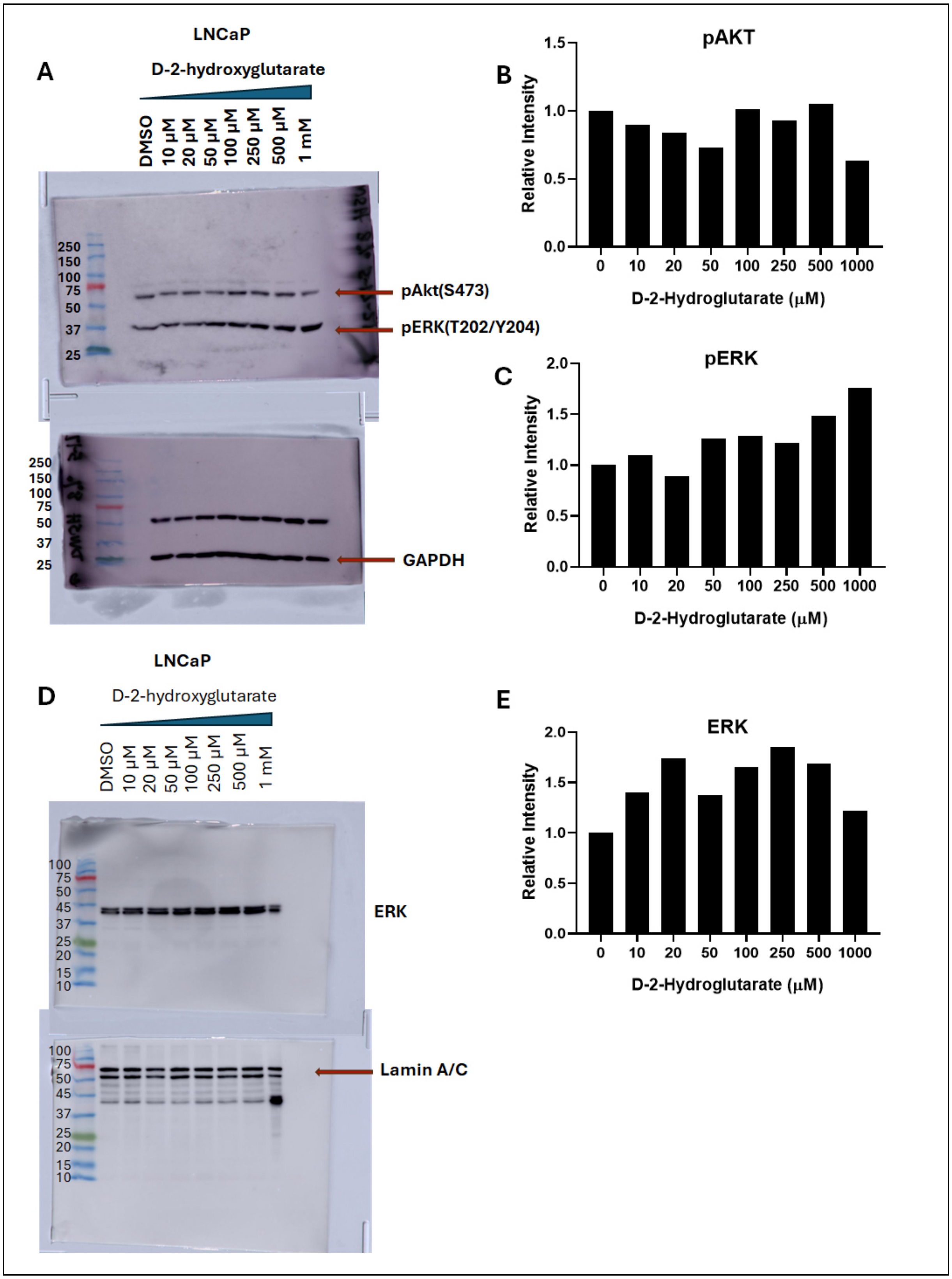

Supplement: Supplementary file 1 [file molecules-30-03316-s001.zip › molecules-3733682-supplementary.pdf]
